# Functional Role of COP1 Gene in Hepatocellular Carcinoma Lipid Metabolism and Stemness

**DOI:** 10.1111/gtc.70108

**Published:** 2026-04-06

**Authors:** Eun‐Hye Jeon, An‐Na Bae, Hajin Lee, Keon Uk Park, Hyun Mu Shin, Jong Ho Park, Kwang Seok Kim, In‐Chul Park, Yun‐Han Lee

**Affiliations:** ^1^ Department of Molecular Medicine Keimyung University School of Medicine Daegu Republic of Korea; ^2^ Department of Anatomy Keimyung University School of Medicine Daegu Republic of Korea; ^3^ Department of Internal Medicine Keimyung University School of Medicine Daegu Republic of Korea; ^4^ Department of Biomedical Sciences Seoul National University College of Medicine Seoul Republic of Korea; ^5^ BK21 FOUR Biomedical Science Project Seoul National University College of Medicine Seoul Republic of Korea; ^6^ Medical Research Center Seoul National University College of Medicine Seoul Republic of Korea; ^7^ Wide River Institute of Immunology Seoul National University Hongcheon Republic of Korea; ^8^ Division of Radiation Biomedical Research Korea Institute of Radiological & Medical Sciences Seoul Republic of Korea

**Keywords:** AKR1D1, COP1, HCC, lipid metabolism, stemness, TMEM65

## Abstract

We have previously defined the constitutive photomorphogenic *protein* 1 (COP1) gene as a therapeutic target in hepatocellular carcinoma (HCC). A recent study demonstrated that COP1 induces non‐alcoholic fatty liver disease (NAFLD), a precursor to HCC, in normal hepatocytes and that reducing COP1 expression significantly improves high‐fat diet‐induced hepatic steatosis. Thus, in this study, we investigated if the function of COP1 was associated with HCC metabolism and evolution. Silencing of COP1 expression by a target siRNA significantly suppressed long‐term colony formation in Huh7, HepG2, Huh1, and PLC/PRF/5 HCC cell lines. RNA sequencing of COP1‐silenced Huh7 and HepG2 cells revealed the same directional regulation of 24 (14 up‐ and 10 down‐regulated) genes. Notable molecular alterations were upregulation of AKR1D1 and downregulation of TMEM65, which involves negative regulation of lipid metabolism and promotion of metastasis, respectively. Correlation analysis using GEPIA2 supported inverse relationship between COP1 and AKR1D1 expression and positive relationship between COP1 and TMEM65 expression in HCC clinical samples. Targeting of COP1 reduced fat accumulation and metastatic potential in both HCC parental cells and CD133^+^ liver cancer stem cells. Overexpression of COP1 reversed the phenotypic changes. Collectively, our findings indicate that the COP1 is functionally correlated with HCC lipid metabolism and stemness.

## Graphical abstract

COP1 promotes HCC lipid metabolism and stemness via the functional interaction with AKR1D1 and TMEM65 genes, respectively, indicating its potential as a therapeutic target for treating primary HCC and preventing metastasis and/or recurrence.

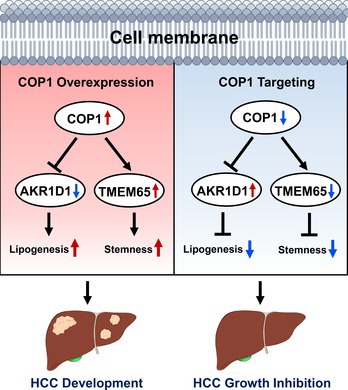

## 1 Introduction

Hepatocellular carcinoma (HCC) is one of the cancers with the highest mortality worldwide, the sixth most diagnosed cancer worldwide, and the third leading cause of cancer‐related deaths (Global Cancer Facts and Figures 2024). According to a recent study, the number of new cases and deaths caused by liver cancer is expected to increase by 55% by 2024 (Rumgay et al. 2022). The major risk factors for HCC include infection with hepatitis B and C viruses, alcohol abuse, obesity, and type 2 diabetes, and exhibit substantial geographic variability (El‐Serag and Rudolph 2007; Michalopoulos 2007). Long‐term exposure to the factors induces the accumulation of genetic and/or epigenetic changes and exploits the hallmarks of cancer, such as reprogramming energy metabolism and activating invasion and metastasis, ultimately leading to tumor evolution in HCC (Feitelson et al. 2002; Roberts and Gores 2005; Thorgeirsson and Grisham 2002; Hanahan 2022; Craig et al. 2020).

Cancer cells use different metabolic pathways to survive and proliferate than normal cells. These metabolic changes, known as the Warburg effect, are characteristic of cancer metabolism (Fukushi et al. 2022). An increase in glycolysis leads to lactic acid accumulation and lowers the pH of the cellular microenvironment (Liberti and Locasale 2016). In addition, cancer cells use fatty acid oxidation because it requires enormous amounts of energy and resources necessary for biosynthesis to maintain rapid growth and proliferation. Fatty acid β‐oxidation plays a crucial role in nonalcoholic fatty liver disease (NAFLD), a precursor to liver cancer, where impaired mitochondrial electron transport chain (ETC) function results in increased β‐oxidation and excessive reactive oxygen species (ROS) production (Chen et al. 2020). Excessive mitochondrial ROS production promotes invasion and migration of cancer cells (Wang et al. 2019). Understanding these metabolic changes is essential for the development of therapeutic strategies that inhibit cancer cell growth and metastasis.

COP1 (also known as RFWD2) gene, an E3‐ubiquitin ligase, regulates proteolysis via the ubiquitin‐proteasome pathway and plays a critical role in various cellular processes. COP1 promotes the ubiquitination of diverse target proteins including wild‐type tumor suppressor p53 (Dornan et al. 2004). These characteristics have significant implications for cell cycle dysregulation and tumor development. COP1 is generally overexpressed in human HCC (Lee et al. 2004). We have previously identified COP1 gene as a therapeutic target in HCC by observing that siRNA knockdown of COP1 restores the levels of tumor suppressors, such as wild‐type p53 and GLIPR1, and induces apoptotic cell death (Lee et al. 2010). Recently, another group found that COP1 causes NAFLD in normal hepatocytes through the degradation of ATGL protein that serves as a major triacylglycerol lipase and controls lipid turnover (Ghosh et al. 2016). This suggests that COP1 plays an important role in regulating liver lipid metabolism and is a potential target for the treatment of metabolic disorders caused by fatty liver disease. Given COP1's role in inducing NAFLD, there are significant questions regarding its involvement in more severe liver pathologies such as HCC. Despite increasing interest in COP1's functions, its specific impact on metabolic processes within HCC cells remains largely unexplored. Based on these previous evidences, we aimed in this study to investigate whether the function of COP1 correlates with HCC metabolism.

## 2 Results

### 2.1 Inhibition of COP1 Expression Upregulates AKRID1 and Downregulates TMEM65 Expression in HCC Cells

**FIGURE 1:**
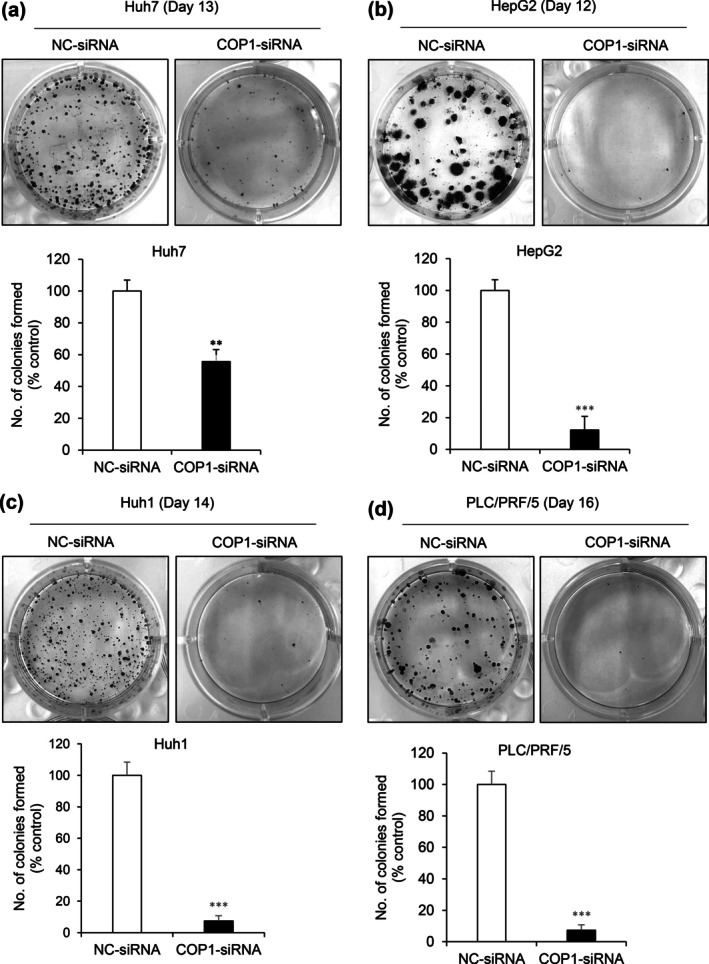
COP1 knockdown inhibits clonogenicity in HCC cells. Representative images and quantitative analysis of colony formation in Huh7 (at day 13) and HepG2 (at day 12) cells treated with NC‐siRNA or COP1‐siRNA. Colony numbers were counted for each cell line. NC‐siRNA, negative control siRNA; COP1‐siRNA, COP1‐specific siRNA. Statistical significance: **p < 0.01; ***p < 0.001 versus control.

**FIGURE 2:**
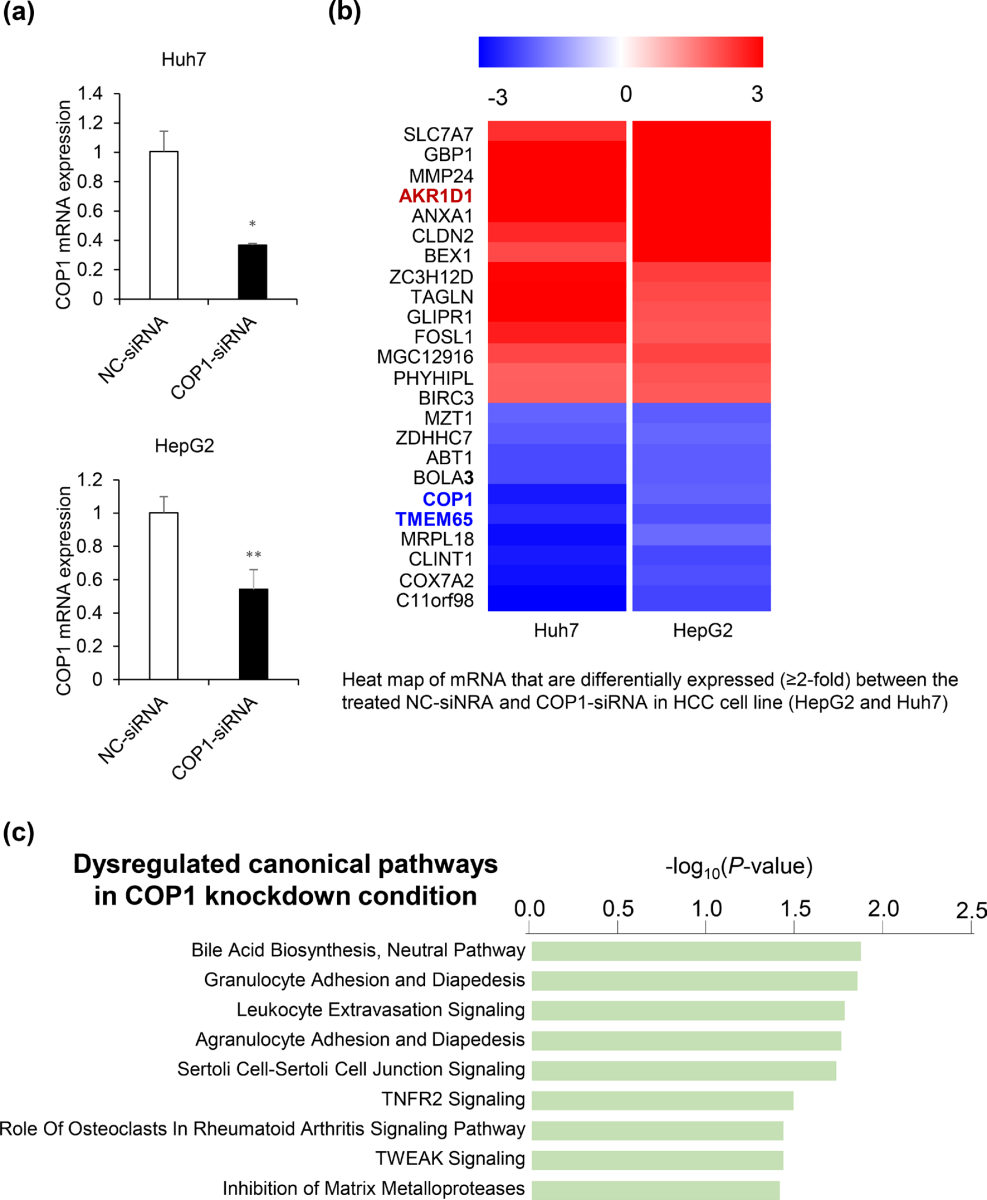
Obtainment of common COP1‐knockdown signature from HCC cells. (a) qRT‐PCR analysis confirming the silencing of COP1 mRNA expression in Huh7 and HepG2 cells with COP1 depletion. Statistical significance: *p < 0.05; **p < 0.01 versus NC‐siRNA. (b) Heat map of 24 genes commonly up‐ or downregulated (≥ 2‐fold change) between NC‐siRNA‐ and COP1‐siRNA‐treated HCC cell lines (Huh7 and HepG2). Red (up‐regulated) and blue (down‐regulated). Specifically, the name of AKRIDI, COP1, and TMEM65 genes are highlighted. (c) IPA canonical pathway analysis of dysregulated genes following COP1 knockdown, with significant enrichment in bile acid biosynthesis and other metabolic pathways.

For this study, we applied the same COP1 target siRNA sequence and transfection condition that were used in our previous study (Lee et al. 2010) and confirmed its reproducibility in terms of HCC growth inhibition. As shown in Figure 1, inhibition of COP1 expression significantly reduced long‐term colony formation in Huh7, HepG2, Huh1, and PLC/PRF/5 HCC cell lines. The target siRNA was transfected into Huh7 and HepG2 HCC cell lines to verify its inhibitory effect on COP1 expression (Figure 2a). After confirming the suppression of COP1 expression, a comprehensive evaluation of the changes in gene expression induced by COP1 inhibition was conducted using RNA sequencing. Sequencing analysis revealed consistent changes across both cell lines, identifying 14 upregulated and 10 downregulated genes (Figure 2b). As a result of functional classification, seven of these genes were associated with metabolic pathways or mitochondrial function, of which three genes (SLC7A7, AKR1D, and PHYHIPL) had increased expression and the remaining four (BOLA3, TMEM65, MRPL18, and COX7A2) had decreased expression (Table 1). These results suggested a close association between COP1 and metabolic gene regulation in HCC cells. Subsequent Ingenuity Pathway Analysis (IPA) showed that the greatest change in bile acid biosynthesis and the neutral pathway was observed with CO1 knockdown (Figure 2c). Specifically, AKR1D1 involves the functional category (Table 2). Steroid hormones and bile acids are potent regulators of hepatic carbohydrate and lipid metabolism. AKR1D1 is highly expressed in hepatocytes where it inactivates steroid hormones and catalyzes an essential step in bile acid synthesis (Nikolaou et al. 2019). Thus, increase in AKR1D1 expression after COP1 silencing suggests that COP1 may negatively affect HCC lipid metabolism. Conversely, TMEM65 expression was downregulated when COP1 expression was inhibited. TMEM65, a mitochondrial inner protein, was known to promote cell mobility and invasiveness in gastric cancer (Shi et al. 2024). When TMEM65 is suppressed by targeting of COP1, a decrease in the migration and invasion of cancer cells may be expected. On the basis of the previous findings, AKR1D1 and TMEM65 genes were focused in this study to reveal COP1's novel role in HCC lipid metabolism and stemness.

**TABLE 1:** List of genes up‐ or down‐regulated in both Huh7 and HepG2 cells with COP1 depletion.

Up‐regulated (14 genes)		
Gene symbol	Full name	Main biological function
SLC7A7	Solute Carrier Family 7 Member 7	Amino acid transporter
GBP1	Guanylate Binding Protein 1	Antiviral and antimicrobial activities
MMP24	Matrix Metallopeptidase 24	Extracellular matrix degradation, neural development
AKR1D1	Aldo‐Keto Reductase Family 1 Member D1	Steroid and bile acid metabolism
ANXA1	Annexin A1	Anti‐inflammatory activity, regulation of cell proliferation
CLDN2	Claudin 2	Tight junction formation, regulation of paracellular permeability
BEX1	Brain Expressed X‐Linked 1	Neuronal differentiation and regeneration
ZC3H12D	Zinc Finger CCCH‐Type Containing 12D	Inflammatory response regulation
TAGLN	Transgelin	Actin binding, regulation of cell shape and motility
GLIPR1	GLI Pathogenesis Related 1	Encodes a protein with similarity to both the pathogenesis‐related protein (PR) superfamily and the cysteine‐rich secretory protein.
FOSL1	FOS like 1, AP‐1 Transcription Factor subunit	Transcriptional regulation, cell proliferation and differentiation
MGC12916	Uncharacterized Protein MGC12916	Predicted protein, function not well defined
PHYHIPL	Phytanoyl‐CoA 2‐Hydroxylase Interacting Protein Like	Function not well defined, possible involvement in lipid metabolism
BIRC3	Baculoviral IAP Repeat Containing 3	Inhibition of apoptosis, regulation of immune response

Down‐regulated (10 genes)		
Gene symbol	Full name	Main biological function
MZT1	Mitotic‐Spindle Organizing Protein Associated with A Ring of Gamma‐Tubulin 1	Spindle assembly, mitotic progression
ZDHHC7	Zinc Finger DHHC‐Type Palmitoyltransferase 7	Protein palmitoylation
ABT1	Activator Of Basal Transcription	Transcriptional regulation
BOLA3	BolA Family Member 3	Mitochondrial iron–sulfur cluster assembly
COP1	Constitutive Photomorphogenic Protein 1	E3 ubiquitin‐protein ligase that mediates ubiquitination and subsequent proteasomal degradation of target protein
TMEM65	Transmembrane Protein 65	Mitochondrial function, cell signaling
MRPL18	Mitochondrial Large Ribosomal Subunit Protein UL18m	Mitochondrial protein synthesis
CLINT1	Clathrin Interactor 1	Vesicle trafficking, clathrin‐mediated endocytosis
COX7A2	Cytochrome C Oxidase Subunit 7A2	Electron transport chain, ATP production
C11orf98	Chromosome 11 Open Reading Frame 98	Function not well defined, potential regulatory role

**TABLE 2:** List of canonical pathways and genes commonly dysregulated in COP1 knockdown condition.

Canonical pathways	−log(*p*‐value)	Molecules (enriched gene expression in COP1 KD vs. SC)
Granulocyte Adhesion and Diapedesis	1.93	CLDN2↑, MMP24↑
Bile Acid Biosynthesis, Neutral Pathway	1.91	AKR1D1↑
Leukocyte Extravasation Signaling	1.86	CLDN2↑, MMP24↑
Agranulocyte Adhesion and Diaoedesis	1.84	CLDN2↑, MMP24↑
Sertoli Cell‐Sertoli Cell Junction Signaling	1.80	CLDN2↑, CLINT1↑
TNFR2 Signaling	1.53	BIRC3↑
Role Of Osteoclasts in Rheumatoid Arthritis Signaling Pathway	1.51	BIRC3↑, MMP24↑
TWEAK Signaling	1.47	BIRC3↑
Inhibition of Matrix Metalloproteases	1.45	MMP24↑
TNFR1 Signaling	1.33	BIRC3↑

*Note:* Pathways and molecules were generated by IPA.

### 2.2 Clinical Relevance of AKR1D1 Upregulation and TMEM65 Downregulation to COP1 Expression

**FIGURE 3:**
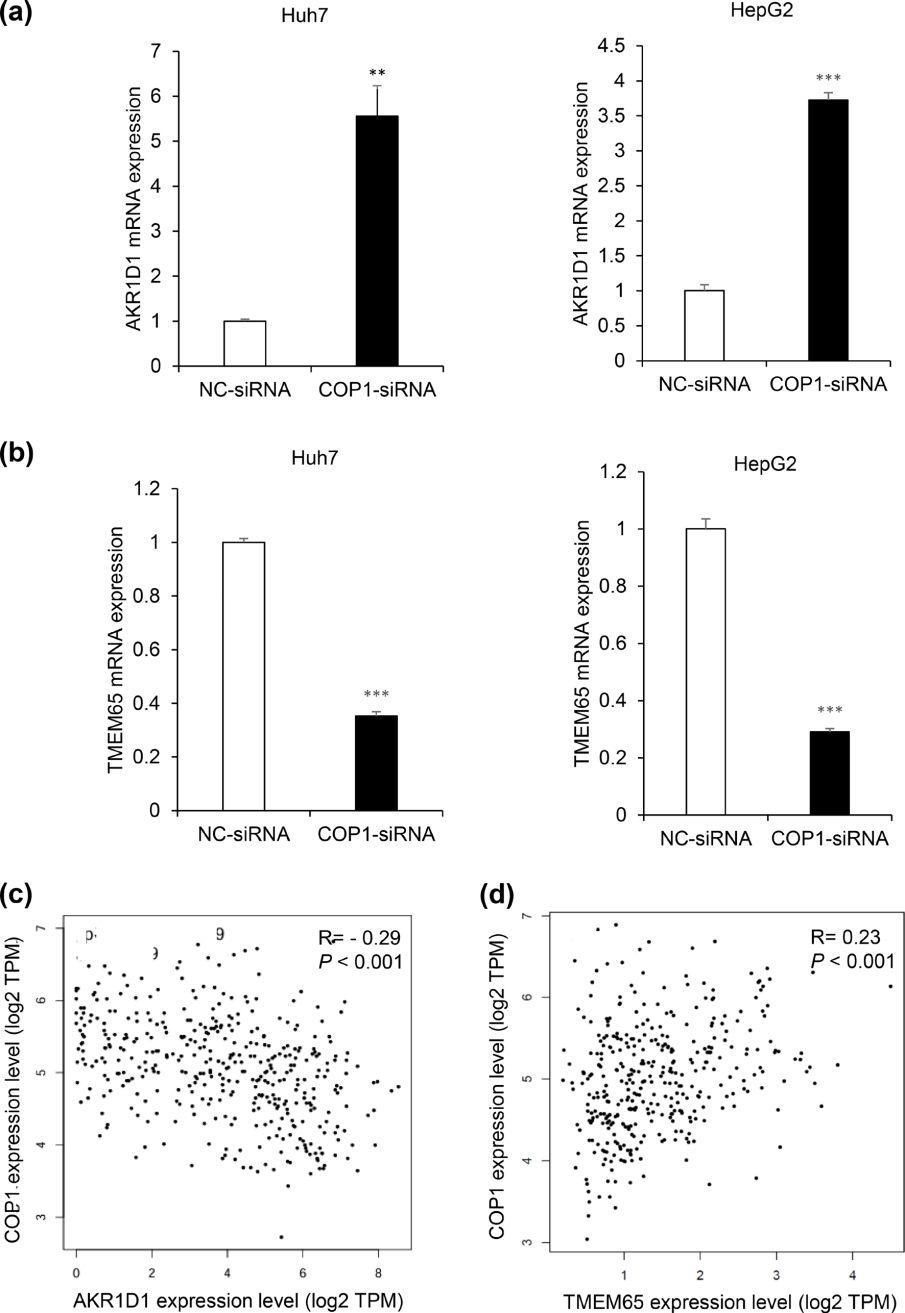
COP1 knockdown upregulates AKR1D1 and downregulates TMEM65 gene expression in HCC cells. (a, b) qRT‐PCR results showing increased AKR1D1 (a) and decreased TMEM65 expression (b) in 48 h of COP1‐siRNA transfection. Statistical significance: **p < 0.01; ***p < 0.001 versus control. (c) Negative correlation between COP1 and AKR1D1 expression in HCC tissues (*R* = −0.29, *p* < 0.001). (d) Positive correlation between COP1 and TMEM65 expression in HCC tissues (*R* = 0.23, *p* < 0.001).

To verify the RNA sequencing results, we performed quantitative real‐time PCR (qRT‐PCR). Consistent with the RNA sequencing results, inhibition of COP1 expression significantly increased AKR1D1 mRNA expression and decreased TMEM65 mRNA expression in both Huh7 and HepG2 cells (Figure 3a,b). This association of differential gene expression was confirmed in the GEPIA2 database. COP1 mRNA expression negatively correlated with AKR1D1 expression (*R* = −0.29, *p* < 0.001) and positively correlated with TMEM65 expression (*R* = 0.23, *p* < 0.001) in HCC patient samples (Figure 3c,d). These findings suggest that the function of COP1 is associated with AKR1D1 and TMEM65 expression for HCC development.

### 2.3 Knockdown of COP1 Impairs Lipid Metabolism and Reduces ROS Levels in HCC

**FIGURE 4:**
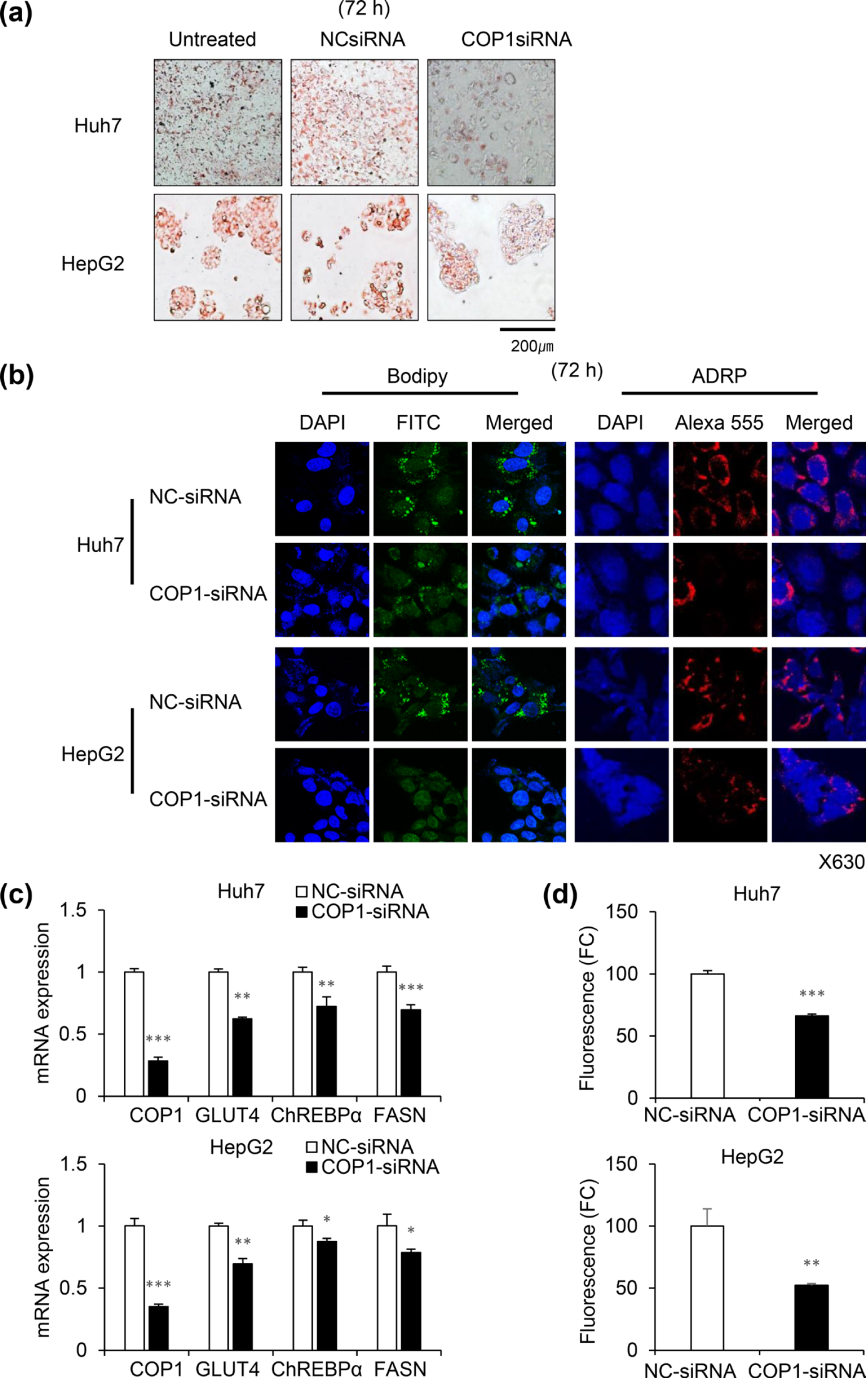
Targeting COP1 decreases lipogenesis, the expression of glucose transporter or fatty acid synthesis genes, and reduces ROS levels in HCC cells. (a‐b) Cells were stained with Oil Red O (a) and fluorescein‐conjugated BODIFY or antibody specific to adipose differentiation‐related protein (ADRP) (b), a marker for lipid droplets (LDs). (c) mRNA levels of genes involved in glucose transport or lipogenesis. GLUT4, glucose transporter 4; FASN, fatty acid synthase N; ChREBPα, carbohydrate response‐element binding protein α. (d) ROS levels in Huh7 and HepG2 cells were measured by fluorescence intensity following COP1 knockdown. Statistical significance: *, *p* < 0.05; **p < 0.01; ***p < 0.001 vs. control.

Dysregulation of fatty acid metabolism and activation of lipogenesis contributes to HCC progression (Wang et al. 2016; Yamashita et al. 2009). After observing the up‐regulation of AKR1D1 gene expression, we next determined to investigate if silencing of COP1 could affect HCC lipid metabolism. As expected, Oil Red O staining confirmed that lipid accumulation was reduced in both Huh7 and HepG2 cells with COP1 depletion (Figure 4a). Lipid droplets (LDs) staining was also performed using the BODIPY and ADRP markers. BODIPYs, which are present inside cells and essential for lipid storage and energy metabolism, were decreased in COP1‐siRNA treated cells compared with NC‐siRNA treated controls (Figure 4b). ADRPs, a protein associated with the surface of lipid droplets, were also decreased in COP1 knockdown cells (Figure 4b). The decrease in lipid droplet formation indicates that COP1 knockdown interferes with lipid synthesis and reduces the lipid storage capacity in the cell, thereby reducing the energy reserves required to support the rapid proliferation and survival of cancer cells. Next, molecular changes underlying the phenotypic change were observed in COP1 knockdown cells. As shown in Figure 4c, silencing of COP1 expression significantly reduced the expression levels of glucose transporter GLUT4 and glucose‐ or insulin‐responsive transcription factor ChREBPα, and the downregulation of the key metabolic factors subsequently reduced the expression of lipogenic enzyme FASN. Reversely, overexpression of COP1 increased the transcription of GLUT4, ChREBPα, FASN, and SREBP1C genes in Huh1 cells, which are essential for glucose or lipid metabolism, leading to increase of short‐term cell proliferation and long‐term colony formation (Figure S1). Reduced lipid metabolism in cancer cells can limit the β‐oxidation of fatty acids, subsequently decreasing substrate availability for the electron transport chain (ETC), where reactive oxygen species (ROS) are generated as byproducts, especially in cancer stem cells (Wang et al. 2019). Thus, the decrease in lipogenesis, as seen in COP1 knockdown cells, is expected to decrease ROS production. As shown in Figure 4d, COP1 knockdown led to a significant reduction in ROS levels in both Huh7 and HepG2 cells compared to the NC‐siRNA controls. These results suggest that COP1 is functionally involved in lipogenesis and liver cancer cell growth.

### 2.4 Targeting of COP1 Suppresses Stemness in HCC

**FIGURE 5:**
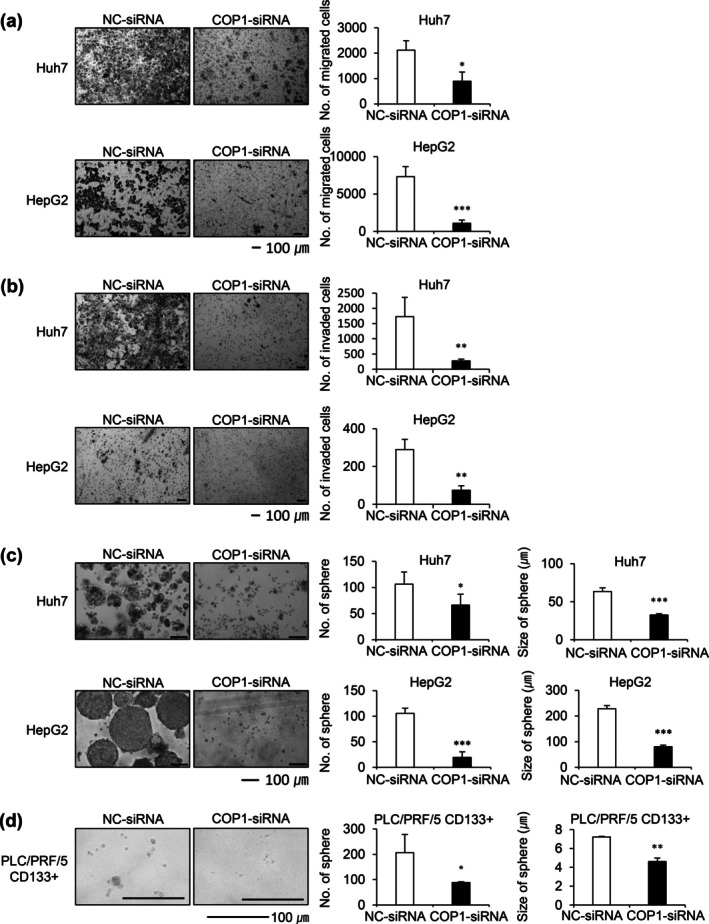
COP1 knockdown reduces motility, and stemness in HCC cells. (a‐b) Migration (a) or invasion (b) assay showing reduced motility in Huh7 and HepG2 cells treated with COP1‐siRNA, as quantified by the relative migration or invaded area. Statistical significance: *, *p* < 0.05; **p < 0.01; ***p < 0.001 vs. control. (c) Sphere formation assay images and quantitative data from Huh7 and HepG2 cells, demonstrating a decrease in both the number and size of spheres in COP1‐siRNA‐treated cells. (d) Sphere formation assay images and quantitative data from PLC/PRF/5 CD133+ cells. Compared to the control grpup, both the sphere number and size decreased upon COP1‐siRNA treatment. Scale bar, 100 μm Statistical significance: *, *p* < 0.05; ***p < 0.001 vs. control.

In addition to decrease in ROS production, TMEM65, a gene known to promote the migration and invasion of certain cancers, was significantly downregulated after COP1 knockdown. Thus, we next determined to observe the broader effects of COP1 knockdown on the metastatic potential of HCC cells. Reflecting those changes, COP1‐siRNA treated cells demonstrated a marked reduction in the migration and invasion ability of the parental HCC cell population (Figure 5a,b). Importantly, COP1 silencing induced a significant decrease in both the number and size of spheres formed in Huh7 and HepG2 cells (Figure 5c). Conversely, Huh1 cells transfected with the COP1‐expressing vector exhibited improved migration ability compared to that of the control vector‐transfected cells (Figure S2). Previous studies identified the CD133 cell surface antigen as a marker of cancer stem/progenitor cells in HCC (Zhu et al. 2010; Song et al. 2008), and we have also reported that upregulation of CD133 contributes to HCC development (Won et al. 2015). Given the functional significance of TMEM65 in metastasis and the observation of TMEM65 repression under COP1 knockdown conditions, we next investigated whether COP1 targeting could affect CRC stemness. To address this, the CD133^+^ CSC population of PLC/PRF/5 cells was sorted from the CD133^−^ population by FACS using an APC‐conjugated CD133 antibody, and successful separation was confirmed by demonstrating that the levels of both CD133 mRNA and protein in the CD133^+^ population were higher than those in the CD133^−^ population (Figure S3). COP1 knockdown significantly reduced the sphere‐forming capacity of CD133^+^ liver CSCs (Figure 5d). This reduction in the sphere‐forming capacity indicates that COP1 knockdown not only disrupts cellular metabolism but also inhibits the tumorigenic and self‐renewal properties of HCC cells, which are crucial for cancer progression and metastasis.

## 3 Discussion

Researchers have spent considerable time developing anticancer drugs through chemotherapy, targeted therapy, and immunotherapy. Recently, metabolic anticancer agents that block the energy source necessary for cancer cell survival following immunotherapy have rapidly emerged as next‐generation treatments. However, because it is still in its infancy, the number of metabolic anticancer is small, and further research is needed to develop more effective metabolic anticancer. Unlike other cancers, liver cancer often develops from chronic liver damage and progresses to cirrhosis, with one major contributing factor NAFLD. As introduced, COP1 is involved in NAFLD progression (Ghosh et al. 2016). Thus, we first aimed in this study if COP1 function was associated with HCC metabolism as well. Notably, the expression of AKR1D1 was commonly upregulated in COP1‐knockdown HCC cells. The inverse correlation between COP1 and AKR1D1 expression in clinical samples was affirmed by a big data analysis. AKRID1 was identified as a significant prognostic marker in HCC samples (Xu et al. 2025). Low expression of AKR1D1 was significantly associated with shorter median survival and advanced TNM clinical stages. Predominantly expressed in the liver, AKR1D1 is involved in the synthesis and breakdown of steroid hormones and bile acids, which are essential for cholesterol metabolism and fat absorption in the intestine. Impaired AKR1D1 function can lead to cholesterol accumulation and bile acid deficiency, affecting membrane integrity and fluidity, which are critical for proliferative cancer cells (Riscal et al. 2019). Fatty acids serve as signaling precursors in metabolic regulation during HCC progression, supporting rapid cell proliferation, survival, invasion, and angiogenesis (Wang et al. 2016). As NAFLD progresses, particularly with advancing fibrosis or inflammation, the expression of AKR1D1 was decreased significantly, playing a crucial role in the pathological progression of NAFLD (Nikolaou et al. 2019). AKR1D1 knockdown increased lipid accumulation and inflammatory responses in the liver. When AKR1D1 expression is reduced, lipid synthesis increases leading to the accumulation of TAG. This accumulation is also associated with inflammation in liver cells. We found in this study that COP1 knockdown reduced the expressions of glucose transport and lipogenesis‐related genes. This supports the hypothesis that inhibition of COP1 may reduce the accumulation of lipid in cancer cells. The reduction of lipid accumulation was confirmed at cellular level as well. The decrease in GLUT4 expression suggests that COP1 knockdown inhibits glucose uptake, thereby reducing glucose utilization in the metabolic processes that are critical for the survival and proliferation of cancer cells. Downregulation of ChREBPα, a transcription factor that regulates lipid and carbohydrate metabolism, suggests that it may disrupt metabolic pathways that support cancer cell proliferation. Decreased levels of FASN, a key enzyme in fatty acid synthesis, indicate impaired membrane biosynthesis and ability to synthesize fatty acids required for energy storage. Collectively, these results show that COP1 knockdown interferes with the overall metabolic activity of cancer cells and potentially inhibiting their growth and survival.

Meanwhile, cancer cells possess metabolic mechanisms that are distinct from those of normal cells, notably the Warburg effect, and obtain energy through fatty acid oxidation to sustain their rapid growth and proliferation (Lee et al. 2022). β‐oxidation, a process in mitochondria and peroxisomes where fatty acids are oxidized to generate energy, naturally produces ROS as a byproduct. The more active the β‐oxidation, the greater the tendency for increased ROS production, occurring during the activation of the mitochondrial ETC. However, if lipid synthesis and accumulation are inhibited due to COP1 suppression, the substrate required for β‐oxidation decreases, likely leading to a reduction in β‐oxidation itself. A decrease in β‐oxidation can lead to reduced ROS production (Adeva‐Andany et al. 2019). NADH and FADH2 produced during β‐oxidation promote ATP synthesis through the ETC, inherently generating ROS. However, if β‐oxidation is reduced due to COP1 inhibition, NADH and FADH2 synthesis also decreases, resulting in a decrease in ROS production produced by the ETC activity. Coinciding with these findings, when the lipid accumulation was reduced by COP1 silencing, mitochondrial ROS production was reduced, suggesting that COP1 inhibition can act as a new mechanism to lower ROS.

In addition to the downregulation of AKR1D1, we have also observed that silencing of COP1 expression induces the downregulation of mitochondrial inner protein TMEM65. TMEM65 upregulation is associated with increased tumor growth, invasion, and migration, as well as poor patient survival, in several specific cancer types including gastric cancer and triple‐negative breast cancer. For example, TMEM65 enhanced gastric cancer cell motility by activating the PI3K‐Akt‐mTOR pathway (Shi et al. 2024) and that decrease in TMEM65 expression disrupted mitochondrial dynamics and reduced ATP production (Zhang et al. 2022). Additionally, it was revealed that excessive ROS production via β‐oxidation promotes metastatic potential in cancer stem cells (Wang et al. 2019). In concordance with those findings, we observed in this study that COP1 knockdown inhibits the abilities of migration, invasion, and sphere forming capacity in HCC cells. Although we originally intended to define the function of COP1 in terms of involvement in HCC metabolism, the correlation of COP1 with cancer stemness was also elucidated throughout RNA sequencing and subsequent diverse experiments. Here we report for the first time that the function of COP1 is associated with both lipid metabolism and stemness in HCC. However, further studies are required to determine whether COP1 regulates the expressions of AKR1D1 and TMEM65 directly or indirectly. This aspect should be explored further in future studies.

## 4 Experimental Procedures

### 4.1 Cell Culture and siRNA Transfection

Human HCC cell lines Huh1, Huh7, HepG2, and PLC/PRF/5 were provided by the Korean Cell Line Bank (KCLB, Seoul, South Korea). All cell lines were incubated in a humidified incubator set to 37°C with 5% CO2. The cells were cultured in DMEM supplemented with 10% fetal bovine serum (FBS) and 1% penicillin/streptomycin (Welgene, Daegu, South Korea). To knock down specific genes in HCC cells, cells were transfected with 20 nM of either negative control (NC) siRNA or COP1‐specific siRNA (#109472; Ambion, Austin, TX, USA) using Lipofectamine 2000 (11668‐019, Invitrogen, Carlsbad, CA, USA) and Opti‐MEM I (31985‐070, Gibco, Waltham, MA, USA) in a humidified incubator for 5 h. The NC‐siRNA was synthesized by Bioneer (Daejeon, South Korea). List of each siRNA sequence is introduced in Table 3.

**TABLE 3:** List of siRNA molecules used in this study.

Name	Target gene (locus ID)	Size (base)	Sequence
COP1‐siRNA	COP1 (64326)	21	S; 5′‐GGAAUGCUUGUCCAAGUUUtt‐3′
		21	AS; 5′‐AAACUUGGACAAGCAUUCCtg‐3′
NC‐siRNA	None	21	S; 5′‐ACGUGACACGUUCGG AGAA(UU)‐3′
		21	AS; 5′‐UUCUCCGAACGUGUCACGU‐3′

Abbreviations: AS, antisense; NC, negative control; S, sense.

### 4.2 Colony Formation Assay

After transfecting HCC cell lines (Huh7, HepG2, Huh1, and PLC/PRF/5) with either NC‐siRNA or COP1‐siRNA, 3 × 10^3^ cells were seeded in six‐well culture plates. Huh7 cells were incubated in a humidified incubator for 13 days and HepG2 cells for 12 days, respectively. Following fixation with methanol, cells were stained with 0.5% crystal violet for 30 min. The colonies were counted under a microscope.

### 4.3 RNA Sequencing and IPA Analysis

The Huh7 and HepG2 liver cancer cell lines were used in this study. Cells were transiently transfected with either NC‐siRNA or COP1‐siRNA for 48 h and harvested for RNA isolation. Total RNA was extracted following standard procedures, and its quality and quantity were assessed prior to RNA sequencing at Macrogen Inc. (Seoul, South Korea). The datasets generated and analyzed in this study are accessible in the GEO database with the accession number GSE287988. Sequencing data were processed to identify differentially expressed genes in COP1‐siRNA‐treated cells compared to the NC‐siRNA controls. Genes with a fold change of ≥ 2.0 and a *p*‐value < 0.05 compared to controls were classified as either upregulated or downregulated. A list of these genes was compiled for each cell line and treatment. Overlapping differentially expressed genes from both Huh7 and HepG2 cell lines treated with COP1‐siRNA were integrated into a common list for further analyses. Heat maps were generated to visually represent changes in gene expression, and IPA was conducted to identify the biological pathways affected by COP1 knockdown. We performed canonical pathway analysis in IPA software (PMID: 24336805) to identify functional characteristics associated with differentially expressed genes (DEGs) in COP1 knockdown.

### 4.4 RNA Extraction and Quantitative Real‐Time PCR (qRT‐PCR)

Changes in gene expression at the mRNA level were evaluated using qRT‐PCR. Total RNA was extracted using the RNeasy Plus Mini Kit (74134; Qiagen, Hilden, Germany). cDNA was synthesized from the extracted RNA using the PrimeScript II 1st strand cDNA Synthesis Kit (Takara Bio, Kusatsu, Japan) according to the manufacturer's instructions. Target genes were amplified using primers synthesized by Genotech (Daejeon, South Korea) and Takara Bio (Table 4).

**TABLE 4:** List of primer sequence of each target gene for qRT‐PCR.

Gene symbol	Full name		Primer sequence
COP1	Constitutive Photomorphogenic Protein 1	Forward	5′‐ACA ATC CCG GTC AAA TTC AA‐3′
		Reverse	5′‐GCA CGT TAG CAT CAA GAC GA‐3′
AKR1D1	Aldo‐Keto Reductase Family 1 Member D1	Forward	5′‐TCA GAA CCT AAA TCG ACC CCT‐3′
		Reverse	5′‐TCC CCA ACT TCG TGT TCA TTT T‐3′
TMEM65	Transmembrane Protein 65	Forward	5′‐TCT ATT GCC ATT GCC CAA G‐3′
		Reverse	5′‐GGA TGA ATA CAT ATC TCA GCT GTC C‐3′
SREBP1C	Sterol Regulatory Element‐Binding Protein 1C	Forward	5′‐CGG AAC CAT CTT GGC AAC AGT‐3′
		Reverse	5′‐CGC TTC TCA ATG GCG TTG T‐3′
CD133	Cluster of Differentiation 133	Forward	5′‐AGT CGG AAA CTG GCA GAT AGC‐3′
		Reverse	5′‐GGT AGT GTT GTA CTG GGC CAA T‐3′
GLUT4	Glucose Transporter Type 4	Forward	5′‐GCC ATG AGC TAC GTC TCC ATT‐3′
		Reverse	5′‐GGC CAC GAT GAA CCA AGG AA‐3′
FASN	Fatty Acid Synthase N	Forward	5′‐AAG GAC CTG TCT AGG TTT GAT GC‐3′
		Reverse	5′‐TGG CTT CAT AGG TGA CTT CCA‐3′
ChREBPɑ	Carbohydrate Responsive Element‐Binding Protein alpha	Forward	5′‐AAG ATC CGC CTG AAC AAC G‐3′
		Reverse	5′‐CAC TTG TGG TAT TCC CGC ATC‐3′
GAPDH	Glyceraldehyde‐3‐Phosphate Dehydrogenase	Forward	5′‐ACA TCA AGA AGG TGG TGA AG‐3′
		Reverse	5′‐GGT GTC GCT GTT GAA GTC‐3′

### 4.5 Correlation Analysis of COP1 and AKR1D1 or TMEM65 Expression in HCC Patient Samples

To assess the correlation between COP1 and AKR1D1 or TMEM65 expression levels in HCC, data were obtained using the Gene Expression Profiling Interactive Analysis (GEPIA2) platform. GEPIA2 was used to extract and visualize transcriptomic data, specifically focusing on the expression levels of COP1 and AKR1D1 or TMEM65 in HCC samples. Expression levels were log_2_‐transformed to normalize transcript counts per million (log_2_ TPM). Pearson's correlation analysis was performed to evaluate the relationship of expression in HCC. The correlation coefficient (*R*) and *p*‐value were computed and displayed on the plot.

### 4.6 Fatty Acid Loading and Oil Red O Staining

Cells were seeded at 1 × 10^5^ cells per well in a six‐well plate containing DMEM and transfected with either NC‐siRNA or COP1‐siRNA for 48 h and then treated with oleic acid at concentrations of 0.25 mM or 0.5 mM for 24 h. The cells were fixed with 4% (w/v) paraformaldehyde for 30 min, rinsed three times with dH_2_O, and incubated for 5 min in 60% isopropanol. The cells were stained for 40 min with Oil Red O solution, washed three times with dH_2_O, and observed under a microscope.

### 4.7 LD Staining

Cells cultured on glass coverslips are used for the methods described below. Cells were transfected with either NC‐ or COP1‐siRNA and washed with PBS 72 h post‐transfection. Cells were fixed with 3% paraformaldehyde for 45 min, followed by three washes with PBS. The cells were permeabilized with 0.1% saponin in PBS for 10 min and then with 0.5% saponin in PBS for another 10 min. To prevent nonspecific binding, cells were treated with 10% normal goat serum (Jackson Laboratory, Bar Harbor, ME, USA) in PBS‐T (containing 0.2% Tween‐20) for 20 min. After three washes in PBS, cells were incubated for 1 h with 100 μL of mouse anti‐human ADRP antibody solution (PROGEN, Heidelberg, Germany). The cells were washed three times in PBS and incubated for 1 h with fluorescein‐conjugated goat anti‐mouse IgG1‐488 antibody diluted in PBS‐T with 2.5% goat serum. Antibody reactions were conducted in a humidified dark environment. After three washes with PBS, the slides were mounted with the ProLong Gold anti‐fade reagent (Invitrogen).

### 4.8 ROS Assay

HCC cell lines (Huh7 and HepG2) transfected with NC‐ or COP1‐siRNA were seeded at a density of 2 × 10^4^ cells/well in black 96‐well plates (SPL Life Sciences, 30296, Pocheon, South Korea). After 24 h, ROS levels were measured using a DCFDA/H2DCFDA‐Cellular Reactive Oxygen Species Detection kit (AB113851, Abcam), according to the manufacturer's instructions. The fluorescence resulting from ROS levels was measured using an Asys UVM 340 microplate reader (Biochrom, Cambridge, UK) at excitation and emission wavelengths of 485/535 nm.

### 4.9 Migration and Invasion Assay

Huh7 and HepG2 cells transfected with either NC‐siRNA or COP1‐siRNA were seeded into the upper chamber of a Transwell migration assay kit (3422; Corning, NY, USA) for the Migration assay or into 24‐well Matrigel invasion chambers (354480; Corning) for the Invasion assay, at a density of 1 × 10^5^ cells per well in 200 μL of DMEM. The lower chamber was filled with 600 μL of DMEM supplemented with 10% FBS. After 72 h of incubation, cells were washed with DPBS, fixed with methanol for 15 min, and washed again with DPBS. The cells were then stained with 0.5% crystal violet for 1 h, followed by multiple washes with DPBS. Images were captured at 100× magnification using a light microscope.

### 4.10 Sphere Forming Assay

Huh7 and HepG2 cells were seeded at 5 × 10^3^ cells per well in 400 μL of medium in a 24‐well plate (Corning). The medium was comprised of DMEM F/12, B27 (50×) (2%, 20 ng/mL), hEGF (20 ng/mL), hbFGF (10 ng/mL), and insulin (4 ng/mL). The cells were then transfected with either NC‐siRNA or COP1‐siRNA using Lipofectamine RNAiMAX (Invitrogen) according to the manufacturer's protocol. The size and number of spheres were measured at 14 days post‐transfection.

### 4.11 Magnetic Cell Sorting

PLC/PRF/5 cell were washed with DPBS and then harvested by detaching the adherent cells using trypsin. The cells were treated with FcR Blocking Reagent and CD133 MicroBeads (130‐100‐857, Miltenyi Biotec, Bergisch Gladbach, Germany) according to the manufacturer's instructions and incubated for 15 min at 4°C in the dark. The labeled cells were separated using the autoMACS Pro system (Mltenyi Biotec).

### 4.12 Statistical Analysis

All experiments were performed at least in triplicate, and all data were statistically analyzed using Microsoft Excel 2016 (Microsoft, Redmond, WA, USA). Statistical evaluation was conducted using Student's *t*‐test, and the results are presented as the mean ± standard error of the mean (SEM). Statistical significance is indicated by *p*‐values with *p < 0.05, **p < 0.01, and ***p < 0.001.

## Author Contributions

**Eun‐Hye Jeon:** validation, investigation, data curation, writing – original draft, visualization. **An‐Na Bae:** formal analysis, investigation, data curation. **Hajin Lee:** formal analysis, investigation, data curation. **Keon Uk Park:** validation, investigation. **Hyun Mu Shin:** formal analysis, investigation. **Jong Ho Park:** validation, investigation. **Kwang Seok Kim:** validation, investigation. **In‐Chul Park:** conceptualization, resources, writing – original draft, supervision, project administration. **Yun‐Han Lee:** conceptualization, writing – original draft, writing – review and editing, supervision, project administration, funding acquisition.

## Funding

This work was supported by the institute for Cancer Research Keimyung University Dongsan Medical Center in 2021.

## Conflicts of Interest

The authors declare no conflicts of interest.

## Supporting information


**Data S1:** Supporting Information.
**Figure S1:** Overexpression of COP1 increases the expression of metabolism‐related genes and HCC cell growth. (a, b) Comparison of COP1 mRNA (a) and protein level (b) in between Huh1 cells transfected with an empty control vector (pCMV6‐Entry Vector) and COP1‐expressiong vector (COP1 Vector). (c), Comparison of mRNA levels of glucose transport or lipogenesis‐related genes in between Huh1 cells transfected with an empty control vector and COP1‐expressiong vector. (d, e), Changes in short‐term cell proliferation (d) and long‐term colony formation after COP1 overexpression. *p < 0.05; **p < 0.01; ***p < 0.001.
**Figure S2:**. Overexpression of COP1 increases the ability of parental HCC cell migration. Huh1 cells (an empty control vector and COP1‐expressiong vector.) were grown up to 100% confluence, scratched, and then wound closure was monitored at 0, 48, 72, and 96 h, respectively. Representative light microscopy images were obtained, and wound closure percentages were calculated and graphed by setting the wound width at 0 h as 0%. **p < 0.01; ***p < 0.001.
**Figure S3:** Separation of CD133^+^ CSCs from CD133^−^ cancer cells in PLC/PRF/5 subculture. After separation, both CD133 mRNA level and protein level were detected for comparison. ***p < 0.001.

## Data Availability

The RNA‐sequencing data of COP1 knockdown Huh7 and HepG2 cell samples are currently available at the GEO database with the accession number of GSE287988.
