# Supplementary material for: Functional Role of COP1 Gene in Hepatocellular Carcinoma Lipid Metabolism and Stemness
Source: Genes Cells. 2026 Apr 6;31(3):e70108. doi: 10.1111/gtc.70108 (PMC13051525; doi:10.1111/gtc.70108)
Supplement: Supplementary file 1 — Data S1: Supporting Information. Figure S1: Overexpression of COP1 increases the expression of metabolism‐related genes and HCC cell growth. (a, b) Comparison of COP1 mRNA (a) and protein level (b) in between Huh1 cells transfected with an empty control vector (pCMV6‐Entry Vector) and COP1‐expressiong vector (COP1 Vector). (c), Comparison of mRNA levels of glucose transport or lipogenesis‐related genes in between Huh1 cells transfected with an empty control vector and COP1‐expressiong vector. (d, e), Changes in short‐term cell proliferation (d) and long‐term colony formation after COP1 overexpression. *p < 0.05; **p < 0.01; ***p < 0.001. Figure S2:. Overexpression of COP1 increases the ability of parental HCC cell migration. Huh1 cells (an empty control vector and COP1‐expressiong vector.) were grown up to 100% confluence, scratched, and then wound closure was monitored at 0, 48, 72, and 96 h, respectively. Representative light microscopy images were obtained, and wound closure percentages were calculated and graphed by setting the wound width at 0 h as 0%. **p < 0.01; ***p < 0.001. Figure S3: Separation of CD133+ CSCs from CD133− cancer cells in PLC/PRF/5 subculture. After separation, both CD133 mRNA level and protein level were detected for comparison. ***p < 0.001. [file GTC-31-0-s001.docx]

**Supplementary Materials**

**Supplementary Figures**

**Supplementary Figure S1**

**
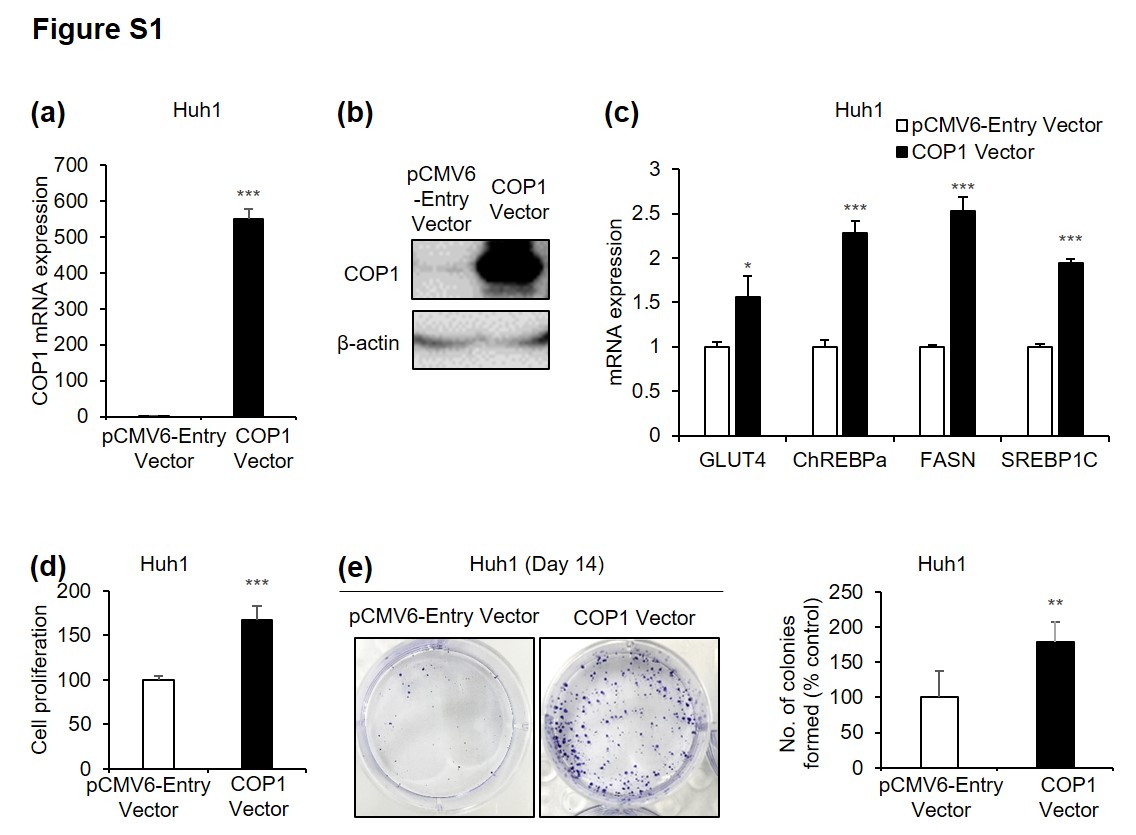
**

**Figure S1.** Overexpression of COP1 increases the expression of metabolism-related genes and HCC cell growth. (a, b) Comparison of COP1 mRNA (a) and protein level (b) in between Huh1 cells transfected with an empty control vector (pCMV6-Entry Vector) and COP1-expressiong vector (COP1 Vector). (c), Comparison of mRNA levels of glucose transport or lipogenesis-related genes in between Huh1 cells transfected with an empty control vector and COP1-expressiong vector. (d, e), Changes in short-term cell proliferation (d) and long-term colony formation after COP1 overexpression. *, *P*<0.05; **, *P*<0.01; ***, *P*<0.001.

**Supplementary Figure S2**


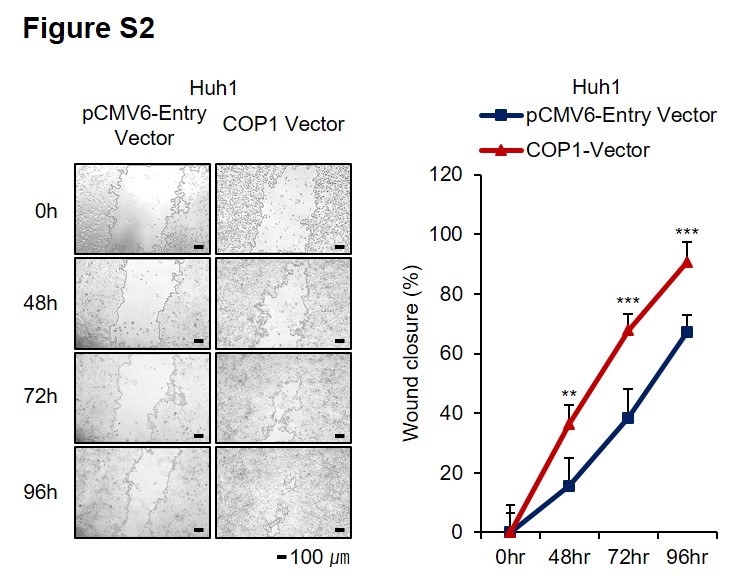


**Figure S2.** Overexpression of COP1 increases the ability of parental HCC cell migration. Huh1 cells (an empty control vector and COP1-expressiong vector.) were grown up to 100% confluence, scratched, and then wound closure was monitored at 0 h, 48 h, 72 h, and 96 h, respectively. Representative light microscopy images were obtained, and wound closure percentages were calculated and graphed by setting the wound width at 0 h as 0%. **, *P*<0.01; ***, *P*<0.001.

**Supplementary Figure S3**

**
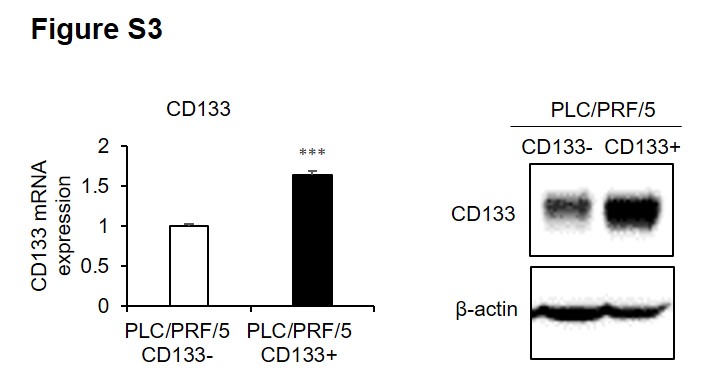
**

**Figure S3.** Separation of CD133^+^ CSCs from CD133^-^ cancer cells in PLC/PRF/5 subculture. After separation, both CD133 mRNA level and protein level were detected for comparison. ***, *P*<0.001.

**Supplementary Materials and Methods**

**Protein extraction and western blotting**

CD133+ PLC/PRF/5 cancer stem cells extracted protein using RIPA buffer (89900, Thermo Fisher Scientific, Waltham, MA, USA) and a Protease and Phosphatase inhibitor cocktail (1861281, Thermo Fisher Scientific). A total of 60ug of protein quantified using the Pierce BCA Protein Assay Kit (23225, Thermo Fisher Scientific) was separated by size using SDS-PAGE on an 8% gel. The protein were then transferred onto a polyvinylidene difluoride (PVDF) membrane (03010040001, Roche, Basel, Switzerland). After blocking the membrane with 5% skim milk (MB-S1667, MB Cell Bio), it was incubated overnight at 4℃ with primary antibody CD133 (64326, Cell signaling Technology, Danvers, MA, USA), COP1 (ab56400, Abcam, Cambridge, UK) and β-actin (sc-47778, Santa Cruz Biotechnology, TA, USA) using Can Get Signal (NKB101, TOYOBO, Tokyo, JAPAN). The membrane as also incubated with secondary antibody goat anti-mouse IgG (115-035-062, Jackson ImmunoResearch Laboratories, West Grove, PA, USA) and goat anti-rabbit IgG (sc-2301, Santa Cruz Biotechnology) using Can Get Signal for 1h at room temperature according to the manufacturer’s instructions. Immunereactive bands were visualized using the Fusion Fx7 imaging system (Vilber Lourmat, Collegien, France).

**Generation of COP1-overexpressing Huh1**

Huh1 cells were transfected with 5ng of either pCMV6-Entry Vector (PS100001, OriGene, Rockville, MD, USA) or a COP1-expressing Vector (RC210492, OriGene) using Lipofectamine 2000 (11668-019, Invitrogen, Carlsbad, CA, USA) and Opti-MEM (31985-070, Gibco, Waltham, MA,USA) according to the manufacturer’s instructions.

**Cell proliferation assay**

The day after transfection of Huh1 cells with either the pCMV6-Entery Vector or the COP1 Vector, Huh1 cells were seeded into 96-well plates (30096, SPL, Korea) at a density of 1x10^3^ cells per well. After 96h, 90ul of DMEM plain media (LM 001-05, WELGENE, Korea) and 10ul of MTT reagent (298-93-1, Duchefa BioChemie, Netherlands) were added to each well, followed by incubation for 1 hour in a humidified incubator. After removing the MTT reagent, DMSO (D2650-100ML, SIGMA, St, USA) was added, and absorbance was measured at 540 nm using an Asys UVM 340 microplate reader (Biochrom, Cambridge, UK).

**Wound healing assay**

The day after transfection of Huh1 cells with either the pCMV6-Entery Vector or the COP1 Vector, 5x10^5^ cells were seeded into 24-well plates (30024, SPL). After 24h of incubation, the monolayer was scratched using 200ul sterile pipette tip. Cell images were captured under a microscope at 100x magnification at 0h, 48h, 72h and 96h. Wound closure was analyzed and quantified using Image J software (National Institutes of Health; NIH, Bethesda, MA, USA).
